# Zika Virus RNA Replication and Persistence in Brain and Placental Tissue 

**DOI:** 10.3201/eid2303.161499

**Published:** 2017-03

**Authors:** Julu Bhatnagar, Demi B. Rabeneck, Roosecelis B. Martines, Sarah Reagan-Steiner, Yokabed Ermias, Lindsey B.C. Estetter, Tadaki Suzuki, Jana Ritter, M. Kelly Keating, Gillian Hale, Joy Gary, Atis Muehlenbachs, Amy Lambert, Robert Lanciotti, Titilope Oduyebo, Dana Meaney-Delman, Fernando Bolaños, Edgar Alberto Parra Saad, Wun-Ju Shieh, Sherif R. Zaki

**Affiliations:** Centers for Disease Control and Prevention, Atlanta, Georgia, USA (J. Bhatnagar, D.B. Rabeneck, R.B. Martines, S. Reagan-Steiner, Y. Ermias, L.B.C. Estetter, T. Suzuki, J. Ritter, M.K. Keating, G. Hale, J. Gary, A. Muehlenbachs, T. Oduyebo, D. Meaney-Delman, W. Shieh, S.R. Zaki);; Centers for Disease Control and Prevention, Fort Collins, Colorado, USA (A. Lambert, R. Lanciotti);; Patología Hospital Universitario de Neiva, Neiva, Colombia (F. Bolaños);; Instituto Nacional de Salud, Bogota, Colombia (E.A. Parra Saad)

**Keywords:** Zika virus, RT-PCR, in-situ hybridization, replication, formalin-fixed, paraffin-embedded tissues, brain, placenta, viruses, vector-borne infections

## Abstract

Zika virus is causally linked with congenital microcephaly and may be associated with pregnancy loss. However, the mechanisms of Zika virus intrauterine transmission and replication and its tropism and persistence in tissues are poorly understood. We tested tissues from 52 case-patients: 8 infants with microcephaly who died and 44 women suspected of being infected with Zika virus during pregnancy. By reverse transcription PCR, tissues from 32 (62%) case-patients (brains from 8 infants with microcephaly and placental/fetal tissues from 24 women) were positive for Zika virus. In situ hybridization localized replicative Zika virus RNA in brains of 7 infants and in placentas of 9 women who had pregnancy losses during the first or second trimester. These findings demonstrate that Zika virus replicates and persists in fetal brains and placentas, providing direct evidence of its association with microcephaly. Tissue-based reverse transcription PCR extends the time frame of Zika virus detection in congenital and pregnancy-associated infections.

Zika virus has recently caused global concern because of an unprecedented outbreak of infection in Brazil and its association with congenital microcephaly and other adverse pregnancy outcomes, including pregnancy loss (*1**–**4*). Vertical transmission of Zika virus from infected mothers to fetuses has been reported (*5**–**7*). However, the mechanism of intrauterine transmission of Zika virus, cellular targets of viral replication, and the pathogenesis that leads to microcephaly and other congenital malformations have not yet been completely elucidated.

Recent in vitro studies that used brain organoids, neurospheres, and human pluripotent stem cell–derived brain cells have demonstrated Zika virus infection of human neural stem and progenitor cells and have also shown that placental macrophages are permissive to Zika virus infection (*8**–**13*)*.* Several studies that used mouse models have revealed that Zika virus infection of mice during early pregnancy results in infection of placenta and fetal brain, causing intrauterine growth restrictions, spontaneous abortions, and fetal demise (*14**–**16*). Animal models and in vitro studies, although providing valuable insights, might not exactly reflect Zika virus disease processes in humans (*9**,**17*)*.* We previously detected Zika virus antigens in placentas of women and in human fetal or neonatal brains (*18**,**19*)*.* However, the presence of antigens does not necessarily indicate virus replication. Previous case studies have detected Zika virus RNA by reverse transcription PCR (RT-PCR) in fetal or neonatal brains, in amniotic fluid, and in placentas of women who had acquired Zika virus infection during early pregnancy (*5**,**20**–**22*)*.* Nevertheless, localization of replicating Zika virus RNA directly in the tissues of patients with congenital and pregnancy-associated infections is critical for identifying cellular targets of Zika virus infection and virus persistence in various tissues and for further investigating the mechanism of Zika virus intrauterine transmission.

Furthermore, laboratory diagnosis of congenital and pregnancy-associated Zika virus infections, particularly those involving adverse pregnancy outcomes, is also challenging because of the typically short duration of viremia (*23**,**24*). Generally, Zika virus RT-PCR can detect viral RNA in serum within 3–10 days of symptom onset (*24**,**25*). Thus, diagnosis by serum RT-PCR can be difficult for neonates who acquire Zika virus infection in utero and for women who acquire (undiagnosed) Zika virus infection during early pregnancy and later experience adverse pregnancy or birth outcomes, because Zika virus RNA generally clears from maternal/infant serum by the time the infant is born or infection is suspected. Serologic testing by ELISA, along with plaque-reduction neutralization testing, can be useful for these cases but may not always provide conclusive Zika virus diagnosis for patients with previous flavivirus exposure or immunization (*23**–**25*) and cannot characterize the virus strain and genotype. As a part of the ongoing Zika virus public health response, we developed Zika virus RT-PCR and in situ hybridization (ISH) assays for the detection and localization of Zika virus RNA in formalin-fixed, paraffin-embedded (FFPE) tissues and tested various tissues from infants with microcephaly who died. We also tested placental/fetal tissues from a series of women suspected of being infected with Zika virus during various stages of pregnancy.

## Methods

### Clinical Specimens

As part of an ongoing public health response effort, we tested FFPE tissue specimens from 52 case-patients (8 infants with microcephaly who died and 44 women) for whom Zika virus infection was clinically and epidemiologically suspected. The tissue specimens were submitted during December 2015–July 2016 to the Centers for Disease Control and Prevention (CDC), National Center for Emerging and Zoonotic Infectious Diseases, Division of High-Consequence Pathogens and Pathology, Infectious Diseases Pathology Branch (Atlanta, GA, USA), by local and state health departments and pathologists for diagnostic consultation. In this series, the definition of a case-patient was 1) a pregnant woman with possible Zika virus disease (based on >1 of the following symptoms: fever, rash, arthralgia, or conjunctivitis and a history of residing in or traveling to countries with active Zika virus mosquito-borne transmission) or 2) an infant with clinical and epidemiologic evidence of possible Zika virus–associated congenital microcephaly. Case-patients were from the United States (n = 38, including 6 from US territories), Brazil (n = 7), and Colombia (n = 7). Tested specimens were placenta, umbilical cord, fetal tissues (from pregnancy loss) from 44 women suspected of being infected with Zika virus during pregnancy, and different portions of the brain (including cerebral cortex, pons, medulla), kidney, liver, spleen, lung, and heart from 8 infants with microcephaly who died. We also included in this analysis all available clinical, demographic, and travel history information and other relevant laboratory results from the state and local health departments and the CDC National Center for Emerging and Zoonotic Infectious Diseases, Division of Vector-borne Diseases, Arboviral Diseases Branch (including available serology and serum RT-PCR results). Clinical status of the infant, including determination of microcephaly or apparently healthy status, was based on the information (generally including anthropometric measurements, physical examination, hearing test and imaging findings) provided to CDC by the state and local health departments and referring clinicians or pathologists as of date of testing. All samples and associated medical and autopsy records were provided in the context of diagnostic consultation, a routine public health service provided by CDC. As such, institutional review was not required for the testing described in this article. Clinical and pathologic findings for 5 cases from Brazil have been previously described (*18**,**19*).

### RNA Extraction, RT-PCR, and Sequencing

We designed 2 sets of primers that target the nonstructural 5 (NS5) and envelope (E) genes of Zika virus and developed RT-PCR assays for the detection of Zika virus RNA from FFPE tissues. We validated the RT-PCR assays by using various positive and negative controls. Positive controls were RNA extracted from FFPE blocks of cultured cells infected with Zika virus prototype (MR766, 1947) and Brazil 2015 strains. Negative controls were RNA extracted from FFPE cell culture controls or tissue specimens from persons with previously confirmed infection with the following viruses: dengue types 1–4, West Nile, yellow fever, Japanese encephalitis, St. Louis encephalitis, eastern and western equine encephalitis, chikungunya, herpes, parvovirus B19, cytomegalovirus, adenovirus, enterovirus, rubella, Powassan, and Lacrosse. We extracted RNA from FFPE tissues of all 52 case-patients (multiple FFPE tissue blocks per patient) by using an optimized extraction protocol as previously described (*26*) and tested the samples by newly developed Zika virus NS5 and E-gene RT-PCR and by RT-PCR for dengue and chikungunya viruses (*27**,**28*). RT-PCR assays were performed by using a QIAGEN OneStep RT-PCR Kit (Valencia, CA, USA) and 5 μl of RNA template, according to the manufacturer’s instructions. The thermocycling conditions used for Zika virus NS5 gene RT-PCR were as follows: 1 cycle at 50°C for 30 min; 1 cycle at 95°C for 15 min; then 40 cycles of incubation at 94°C, 56°C, and 72°C for 1 min each; followed by 1 cycle of final extension at 72°C for 10 min. The primer sequences, annealing temperatures, and amplification product sizes of the RT-PCRs are summarized in Table 1. The NS5 (127-bp) and E (209-bp) gene–positive amplicons were directly sequenced on a GenomeLab GeXP Genetic Analysis System (AB SCIEX, LLC, Redwood City, CA, USA). The search for homologies to known sequences was performed by using the BLAST nucleotide database (http://blast.ncbi.nlm.nih.gov/Blast.cgi). To evaluate the level of fragmentation and presence of PCR inhibitors, we also tested each sample by housekeeping gene 18S rRNA RT-PCR by using QuantumRNA Classic 18S Internal Standard (Life Technologies, Carlsbad, CA, USA).

**Table 1:** Oligonucleotide primers used for RT-PCR assays*

RT-PCR	Primers		Sequence, 5′→3′	Gene target	Product size, bp	Annealing temperature, °C	Reference
Zika virus	Forward	AAG TAC ACA TAC CAA AAC AAA GTG GT		NS5	127	56	This study
	Reverse	TGT TAA GAG CGT AAG TGA CAA C					
Zika virus	Forward	TGC CCA ACA CAA GGT GAA GC		E	209	58	This study
	Reverse	ACT GAC AGC ATT ATC CGG TAC TC					
DENV1–4	Forward	AAG GAC TAG AGG TTA KAG GAG ACC C		3′ UTR	110	62	(*27*)
	Reverse	GGC GYT CTG TGC CTG GAW TGA TG					
CHIKV	Forward	TCA CTC CCT GTT GGA CTT GAT AGA		PPG	126	55	(*28*)
	Reverse	TTG ACG AAC AGA GTT AGG AAC ATA CC					

*CHIKV, chikungunya virus; DENV, dengue virus; E, envelope; NS, nonstructural; PPG, polyprotein gene; RT-PCR, reverse transcription PCR; UTR, untranslated region.

To calculate the Zika virus RNA copy number in tissues of case-patients positive by conventional RT-PCR, we also performed a quantitative real-time RT-PCR by using primers as described previously (*23*)*.* The amount of human β-actin mRNA in the RNA extracted from each section was also determined and used as an internal reference for normalization. The relative copy number of Zika virus RNA was calculated by using the β-actin mRNA copy number, estimated at 1,500 copies/cell, as previously described (*29*)*.*

### ISH 

Zika virus ISH was performed by using sense and antisense riboprobes that target multiple genes of Zika virus (Advanced Cell Diagnostics, Newark, CA, USA). ISH was developed and validated on Zika virus–positive culture cells and on various Zika virus–negative controls, including tissues from case-patients or cultures positive for dengue virus, West Nile virus, and chikungunya virus. Riboprobes targeting dengue virus and *dap*B gene (Advanced Cell Diagnostics) were also used as negative control probes for ISH. To localize Zika virus genomic RNA (using antisense probe) and negative-sense replicative RNA intermediates (using sense probe) in tissues, we performed ISH on FFPE brain and placental tissues positive for Zika virus by RT-PCR, as previously described (*30*)*.* To examine pathologic changes in the tissue, we also analyzed tissue sections from all case-patients by routine histopathology techniques. To define specific cell types, we performed immunohistochemical analysis by using antibodies against neuronal nuclei (Abcam, Cambridge, MA, USA), glial fibrillary acidic protein (Agilent, Santa Clara, CA, USA), and CD163 (Leica Biosystems, Buffalo Grove, IL, USA) on serial sections of block positive for Zika virus by ISH from selected case-patients, according to previously described protocol (*19*)*.*

### Statistical Analyses

Statistical analyses were performed by using GraphPad Prism statistical software, version 6.0a (Graph Pad Software Inc., La Jolla, CA, USA). We compared demographic and clinical variables between the 2 groups by using the Fisher exact test (2-sided). We used the Mann–Whitney U test for 2-group comparisons of continuous data. Differences were considered statistically significant at p<0.05.

## Results

### Case-Patients Characteristics

We identified Zika virus RNA by RT-PCR in various tissue specimens from 32 (62%) case-patients (Table 2). Median maternal age (age of pregnant women and mothers of infants) was 27 years (range 15–39 years) for case-patients with positive Zika virus RT-PCR results and 29 years (18–43 years) for case-patients with negative Zika virus RT-PCR results; all case-patients had Zika virus infection–like symptoms. For the 32 case-patients with positive Zika virus RT-PCR results, the commonly reported signs were rash (94%), fever (59%), arthralgia (28%), headache (19%), and conjunctivitis (13%). For the 20 case-patients with negative Zika virus RT-PCR results, the most commonly reported signs were rash (80%), fever (45%), arthralgia (40%), headache (20%), and conjunctivitis (20%). No distinctive maternal clinical features between case-patients positive and negative for Zika virus by tissue RT-PCR were identified. Maternal symptom onset occurred during the first trimester for 27 (52%) case-patients and during the second or third trimester of pregnancy for 24 (46%) (Table 2). Information about timing of symptom onset was not available for 1 case-patient. Of 52 case-patients, 30 (58%) had an adverse pregnancy or birth outcome and 22 (42%) had live-born, apparently healthy, infants (Table 2). 

**Table 2:** Symptom onset trimester, pregnancy outcomes and Zika virus tissue RT-PCR results of 52 case-patients*

Pregnancy or infant outcome	No. (%) case-patients, n = 52	Trimester of maternal symptom onset		Zika virus–positive case-patients, by tissue RT-PCR, no. (%), n = 32
		First, no. (%), n = 27	Second or third, no. (%), n = 24	
Spontaneous abortion	11 (21)	11 (41)	NA	9 (82)
Elective termination	3 (6)	3 (11)	NA	3 (100)
Intrauterine fetal demise†	3 (6)	1 (4)	2 (8)	0
Infant with microcephaly (fatal outcome)‡	8 (15)	8 (29)	NA	8 (100)
Infant with microcephaly (nonfatal outcome)§	5 (10)	3 (11)	1 (4)	4 (80)
Apparently healthy infant	22 (42)	1 (4)	21 (88)	8 (36)

*Of 32 case-patients with positive Zika virus tissue RT-PCR results, maternal serology (IgM and plaque-reduction neutralization test) results were consistent with recent flavivirus infection (9 case-patients) and consistent with recent Zika virus infection (4 case-patients). Zika virus infection was confirmed for 5 patients by RT-PCR at a state laboratory. For the remaining 14 case-patients, no maternal testing was performed. Of 20 case-patients with negative Zika virus tissue RT-PCR results, maternal serology (IgM and plaque-reduction neutralization test) results were consistent with recent flavivirus infection for 12 case-patients (11 who had live-born, apparently healthy, infants, and 1 who had intrauterine fetal demise). For 2 case-patients, maternal serology results were negative for Zika virus IgM, and for 6 case-patients, no maternal serologic testing was performed. Zika virus infection was confirmed for 1 case-patient by RT-PCR at a state laboratory. NA, not available; RT-PCR, reverse transcription PCR. †Including 1 with microcephaly. ‡Died postnatally. §Information about timing of symptom onset was unavailable for 1 case-patient with Zika virus–positive tissue RT-PCR results. Nonfatal, according to the information received from the case submitters as of the date of testing.

### RT-PCR and Sequencing 

Brain and placental tissues from 32 (62%) of the 52 case-patients were positive by both Zika virus RT-PCR assays (gene targets NS5 and E). Sequence analysis of all positive amplicons showed 99%–100% nt identities with Zika virus Asian genotype strains currently (2015–2016) circulating in Brazil. RT-PCR results were positive for 24 (75%) of 32 case-patients with adverse pregnancy (n = 12) or birth (n = 12) outcomes and positive for 8 (36%) of 22 case-patients with live-born, apparently healthy, infants (p = 0.0082). Of 24 case-patients with positive RT-PCR results and adverse pregnancy/birth outcomes, 23 had maternal symptom onset during the first trimester, whereas all 8 case-patients with apparently healthy infants and positive RT-PCR results had symptom onset in the third trimester (p<0.0001).

Of the 13 microcephaly-associated case-patients (Table 3), 8 were infants with microcephaly and fatal outcome (died within few minutes to 2 months after birth) and 5 were women who had live-born infants with microcephaly and nonfatal outcome. Of these, 12 (92%) were positive for Zika virus by tissue RT-PCR. Zika virus RNA was detected by RT-PCR in brain tissues of all 8 infants; all had maternal symptom onset during the first trimester. Other tested tissues from infants (kidney, liver, spleen, heart, and rib) were negative by RT-PCR. Of the 17 case-patients with adverse pregnancy outcomes (Table 4), Zika virus RNA was detected by RT-PCR in placentas/umbilical cord/fetal tissues of 12 (70%); all had symptom onset during the first trimester. Of 22 case-patients who had live-born, apparently healthy, infants, including 8 case-patients with positive RT-PCR results, 21 (95%) had symptom onset during the second or third trimester.

**Table 3:** Characteristics and laboratory findings for 13 microcephaly-associated case-patients*

Case-patient no.	Maternal travel history or residence	Maternal symptom onset, gestation wk/trimester	Outcome	End of pregnancy, gestational age, wk/trimester	Results of Zika virus testing performed on FFPE tissues	
					RT-PCR	ISH†
54	Brazil	4/first	Infant with microcephaly died 6 h after birth	38/third	Positive (brain); negative (placenta, spleen, kidney, lung, liver)	Positive (brain)
66	Colombia	8/first	Infant with microcephaly died 2 d after birth	26/third	Positive (brain, placenta); negative (liver)	Positive (brain)
67	Colombia	8/first	Infant with microcephaly died shortly after birth	27/third	Positive (brain, placenta); negative (liver)	Positive (brain)
68	Colombia	10/first	Infant with microcephaly died shortly after birth	27/third	Positive (brain, placenta); negative (liver)	Positive (brain), negative (placenta)
55	Brazil	NA/first	Infant with microcephaly died few min after birth	29/third	Positive (brain); negative (placenta)	Positive (brain)
53	Brazil	NA/first	Infant with microcephaly died 20 h after birth	36/third	Positive (brain); negative (placenta, spleen, kidney, heart)	Positive (brain)
37	Brazil	NA/first	Infant with microcephaly died 60 d after birth	38/third	Positive (brain)	Positive (brain)
65	Colombia	NA/first	Infant with microcephaly died few minutes after birth	28/third	Positive (brain, placenta); negative (liver)	Negative (brain, placenta)
49	Brazil, delivered in USA	7/first	Infant with nonfatal microcephaly	37/third	Positive (placenta)	Tissue NA
83	Cape Verde‡	7/first	Infant with nonfatal microcephaly	36/third	Positive (placenta)	Negative (placenta)
20	Marshall Islands‡	Unknown	Infant with nonfatal microcephaly	31/third	Positive (placenta)	Tissue NA
85	Honduras‡	10/first	Infant with nonfatal microcephaly	37/third	Positive (placenta, umbilical cord)	Negative (placenta)
13	Dominican Republic	18/second	Infant with nonfatal microcephaly	39/third	Negative (placenta, umbilical cord, membrane)	ND

*FFPE, formalin-fixed, paraffin-embedded; ISH, in situ hybridization; NA, not available; ND, not done; RT-PCR, reverse transcription PCR. †ISH results include staining by sense and antisense probes. ‡Travel history.

**Table 4:** Characteristics and laboratory findings of case-patients with adverse pregnancy outcome*

Case-patient no.	Maternal travel history or residence	Maternal symptom onset, gestation wk/trimester	Outcome	End of pregnancy, gestational age, wk/trimester	Results of Zika virus testing performed on FFPE tissues		
					RT-PCR	ISH†
81	Colombia	1/first	SA	6/first	Positive (placenta)	Positive (placenta)
47	Honduras‡	5/first	SA	≈8/first	Positive (placenta)	Positive (placenta)
57	Puerto Rico‡	5/first	SA	8/first^t^	Positive (placenta)	Positive (placenta)
56	Guatemala‡	6/first	SA	11/first	Positive (placenta)	Positive (placenta)
18	American Samoa	7/first	SA	14/second	Positive (placenta, fetal tissue)	Positive (placenta, fetal tissue)
78	Colombia	7/first	SA	11/first	Positive (placenta)	Positive (placenta)
125	Brazil	8/first	SA	11/first	Positive (placenta)	Positive (placenta)
256	Brazil	8/first	SA	13/first	Positive (placenta)	Negative (placenta)
79	Colombia	NA/first	SA	9/first	Positive (placenta)	Positive (placenta)
80	Mexico‡	6/first	SA	12/first	Negative (placenta, cord)	ND
19	Dominican Republic‡	1/first	SA	10/first	Negative (placenta)	ND
45	Honduras‡	13/first	ET	19/second	Positive (placenta); negative umbilical cord, fetal brain, liver, lung)	Positive (placenta)
76	Puerto Rico	7/first	ET	9/first	Positive (placenta)	Negative (placenta)
28	Dominican Republic‡	NA/first	ET	16/second	Positive (placenta)	Negative (placenta)
97	El Salvador	NA/first	IUFD§	34/third	Negative (placenta)	ND
92	American Samoa	NA/second	IUFD	24/second	Negative (placenta, umbilical cord, membrane)	ND
556	Marshall Islands‡	31/third	IUFD	36/third	Negative (placenta, umbilical cord, membrane)	ND

*ET, elective termination; FFPE, formalin-fixed, paraffin-embedded; ISH, in situ hybridization; IUFD, intrauterine fetal demise; NA, not available; ND, not done; RT-PCR, reverse transcription PCR; SA, spontaneous abortion.  †ISH results include staining by both sense and antisense probes.  ‡Travel history. §With microcephaly.

**Figure 1:**
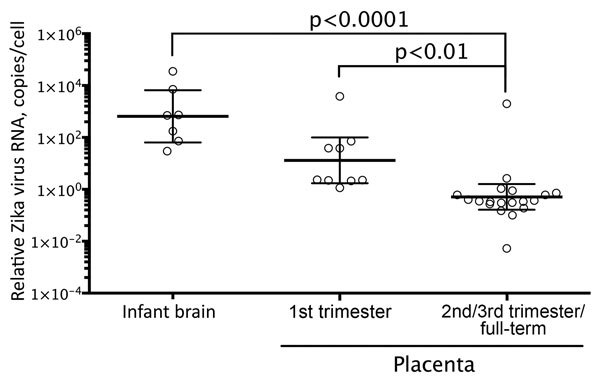
Zika virus RNA load levels in human brain and placental tissues. The scatter plot graph shows the relative levels of Zika virus RNA in formalin-fixed, paraffin-embedded tissue sections, which were quantified by real-time quantitative reverse transcription PCR by using primer-probe sets for Zika virus envelope gene and β-actin mRNA. β-actin mRNA was used as an internal reference gene that provided a normalization factor for the amount of RNA extracted from a section. The copy number of Zika virus RNA per cell was calculated using β-actin mRNA copy number, which was estimated to be 1,500 copies/cell. The graph shows individual data points and superimposed horizontal lines at the geometric mean, and error bars show the 95% CI for that geometric mean. p values were calculated with nonparametric 1-way analysis of variance (Kruskal-Wallis test) followed by Dunn multiple comparison tests. The relative Zika virus RNA copy numbers for second/third trimester or full-term placentas were statistically significantly lower than those for first trimester placentas or infant brain tissues.

The time frame from maternal symptom onset to detection of Zika virus RNA by RT-PCR in brains was 119–238 (mean 163) days and in placentas was 15–210 (mean 81) days. Relative levels of Zika virus RNA in the infant brain tissues (Figure 1) were ≈1,200-fold higher than those in the second or third trimester or full-term placentas (brain geometric mean 651.9 [95% CI 63.91–6,650] copies/cell; second or third trimester or full-term placentas geometric mean 0.5129 [95% CI 0.1649–1.595] copies/cell). In addition, relative levels of Zika virus RNA in the first trimester placentas (13.10 [1.718–99.87] copies/cell) were 25-fold higher than those in the second or third trimester or full-term placentas.

### ISH 

**Figure 2:**
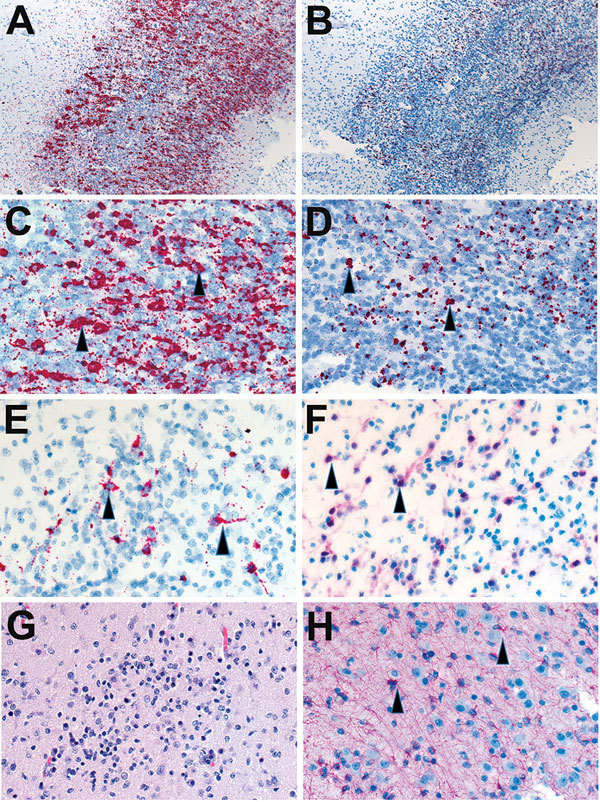
Localization of Zika virus RNA by in situ hybridization in brain tissues from infants with microcephaly. A) ISH with use of antisense probe. Zika virus genomic RNA (red stain) in cerebral cortex of an infant (case-patient no. 66, gestational age 26 wk). Original magnification ×10. B) ISH with use of sense probe. Serial section showing negative-strand replicative RNA intermediates (red stain) in the same areas shown in panel A. Original magnification ×10. C) ISH with use of antisense probe. Higher magnification of panel A, showing cytoplasmic staining of neural (arrowheads) and glial cells. Original magnification ×20. D) ISH with use of sense probe. Higher magnification of panel B, showing cytoplasmic staining of neural and glial cells (arrowheads). Original magnification ×20. E) ISH with use of antisense probe. Localization of negative-strand replicative RNA intermediates in neural cells or neurons (red, arrowheads) of another infant with fatal outcome (case-patient no. 67, gestational age 27 wk). Original magnification ×40. F) Immunostaining of neurons (arrowheads) with use of antibodies against neuronal nuclei in a serial section. Original magnification ×40. G) Hematoxylin and eosin stain showing cortical neural cells in a serial section. Original magnification ×40. H) Immunostaining of glial cells (arrowheads) with use of glial fibrillary acidic protein antibody in the same case. Original magnification ×40. ISH, in situ hybridization.

**Figure 3:**
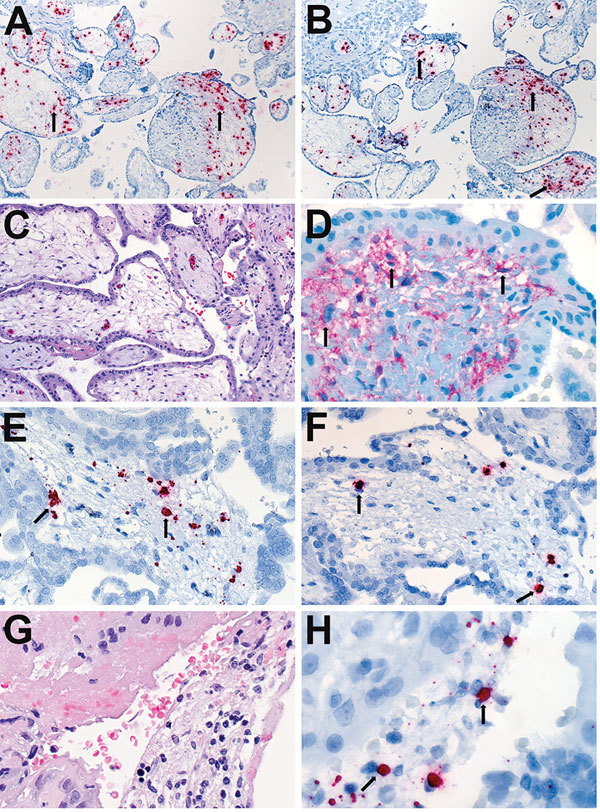
Localization of Zika virus RNA by ISH in placental tissues of women after spontaneous abortion. A) ISH with use of antisense probe. Zika virus genomic RNA localization in placental chorionic villi, predominantly within Hofbauer cells (red stain, arrows), of a case-patient who had spontaneous abortion at 11 wk gestation (case-patient no. 56). Original magnification ×10). B) ISH with use of sense probe. Serial section showing negative–strand replicative RNA intermediates (red stain, arrows) in the same cells shown in panel A. Original magnification ×10. C) Hematoxylin and eosin stain of placental tissue of a case-patient who experienced spontaneous abortion at 8 wk gestation (case-patient no. 47). Original magnification ×20. D) Immunostaining for CD163 highlighting villous Hofbauer cells in a serial section as seen in panel C. Original magnification ×63. E) ISH with use of antisense probe. Zika virus genomic RNA as seen in a serial section from the same case-patient as in panel C, showing staining within Hofbauer cells (red stain, arrows) of placental chorionic villi. Original magnification ×40. F) ISH with use of sense probe. Serial section showing negative-strand replicative RNA intermediates (red stain, arrows) in the same cells as shown in panel E. Original magnification ×40. G) Hematoxylin and eosin stain from the same case-patient as in panel C, showing inflammatory cell infiltrates in maternal side of placenta. Original magnification ×63. H) ISH with use of sense probe. Negative-strand replicative RNA intermediates (red stain, arrows) in inflammatory cells in a serial section. Original magnification ×63. ISH, in situ hybridization.

Overall, Zika virus RNA was demonstrated by ISH in tissues of 16 (50%) of 32 case-patients with positive Zika virus RT-PCR results. Intense signals from antisense (which binds to Zika virus genomic RNA) and sense (which binds to replicative RNA intermediates) probes were observed in brain tissues (Figure 2) of 7 of 8 infants with microcephaly. Zika virus replicative RNA, detected by using sense probe, was observed in the neural cells, neurons, and degenerating glial cells within the cerebral cortex of the brain (Figure 2). Immunostaining with antineuronal nuclei and antiglial fibrillary acidic protein was also noted in the areas of ISH staining (Figure 2). Zika virus genomic and replicative RNA was also localized in placental chorionic villi (Figure 3) of 9 (75%) of 12 women with positive Zika virus RT-PCR results who had an adverse pregnancy outcome during the first or second trimester. All 9 of these women had symptom onset during the first trimester of pregnancy. Zika virus replicative RNA was predominately observed in the Hofbauer cells of the placental chorionic villi, as identified by immunohistochemistry studies with CD163 cell marker (Figure 3). For 6 of 8 women positive for Zika virus by RT-PCR and who had apparently healthy infants, placental tissues were available for ISH testing and were all negative. Zika virus–positive culture cells and tissues from case-patients positive for Zika virus showed no signal when tested by using dengue virus and DapB probes. 

## Discussion

This work demonstrates evidence of Zika virus RNA replication in placentas of women who had pregnancy losses during the first or second trimester of pregnancy and in brain tissues of infants with microcephaly. We directly localized Zika virus negative-sense replicative intermediates in Hofbauer cells of placenta and neural cells and neurons by using ISH. Our findings indicate that Hofbauer cells may play a role in the dissemination or transfer of Zika virus to the fetal brain, particularly during early pregnancy. Furthermore, tissue-based RT-PCR assays described herein also extend the time frame for Zika virus detection; thus, the assays can be a valuable adjunct for the diagnosis of congenital and pregnancy-associated infections and can help to expand diagnostic opportunities for Zika virus, particularly when the mother was not previously tested, the window of detection for serum RT-PCR and serology has passed, or results of Zika virus testing are inconclusive (e.g., serology consistent with recent flavivirus infection). In addition, our findings also reveal the persistence of Zika virus RNA in placenta and brain tissues, which might provide insights into the potential late or long-term sequelae of the infection.

In this series of case-patients, Zika virus clearly exhibited neurotropism. In 7 of 8 infants with microcephaly, the presence of Zika virus genomic and replicative RNA was observed in neural cells, neurons, and degenerating glial cells of cerebral cortex by ISH; whereas, all other tested tissues from these case-patients were negative by RT-PCR and ISH. Thus, our findings support those of previous in vitro and mouse studies, which demonstrated that Zika virus infects human neural stem and progenitor cells and causes severe pathologic changes in the brain but not in other visceral organs (*8**–**11**,**16*). A recent study also reported higher expression of Zika virus entry receptor AXL in radial glia (the neural stem cells of the human fetal cerebral cortex), astrocytes, and neural progenitors (*31*)*.* Furthermore, we noted that the relative levels of Zika virus RNA in the brain tissues of infants were >1,000-fold higher than those in placentas, according to real-time quantitative RT-PCR, which also suggests replication of Zika virus in brain. Previous case reports describe identification of Zika virus antigens and RNA predominately in fetal brain tissues by IHC and RT-PCR (*6**,**18**–**21*)*.* We also noticed that in all microcephaly cases that were positive by tissue RT-PCR, maternal symptom onset occurred during the first trimester, which might suggest that the virus can cause abnormal brain development when infection occurs early in organogenesis. Previous studies of rubella reported that the risk for fetal infection with congenital anomalies is highest when exposure/infection occurred before 11–12 weeks of gestation and sharply decreased with increasing gestational age (*32*)*.*

Findings also demonstrate detection of Zika virus RNA by RT-PCR in 12 (86%) of 14 women who had spontaneous abortions or fetal losses. All of these women had symptom onset during the first trimester. For 9 of these 12 case-patients (all with pregnancy losses at <19 weeks gestational age), replicative Zika virus RNA was demonstrated in Hofbauer cells (placental macrophages) of chorionic villi, suggesting direct infection and replication of Zika virus in Hofbauer cells. We have previously reported the presence of trophoblast necrosis and fibrin deposit along with viral antigens in placenta, which may also indicate placental damage by direct infection (*33*)*.* A recent in vitro study also demonstrated that Zika virus infects and primarily replicates in Hofbauer cells (*13*)*.* Prior studies have identified macrophages as target cells for dissemination of dengue virus as well (*34*)*.* Taken together, these findings suggest that Hofbauer cells, which have access to fetal blood vessels, may facilitate transfer or dissemination of the virus to the fetal brain. Of note, in this case series, Zika virus RNA was not detected in any of the 3 women who had intrauterine fetal demise. Conversely, we detected Zika virus RNA by RT-PCR in placentas of 4 women who had live-born infants with nonfatal microcephaly; however, viral load in the placentas was low. This finding may indicate temporal persistence of Zika virus RNA in placental tissues. Persistence of other viruses in immune-privileged organs (e.g., eyes, placenta, fetal brain) has been reported (*35**,**36*)*.* Previous studies related to other arboviruses, including West Nile and chikungunya viruses, have also reported persistence of arbovirus RNA in various tissues by RT-PCR (*37**–**39*).

Zika virus RNA (low copy number) was also detected in placental tissues of 8 women who had apparently healthy infants. In each of these women, symptom onset began during the third trimester; for 5 of them, serologic evidence of Zika virus or unspecified flavivirus infection was found. However, in 3 apparently healthy infants, serum/cord blood RT-PCR or serology results were negative for Zika virus or flavivirus; for remaining 5 infants, Zika virus testing was not performed. Detection of Zika virus RNA in the placenta by RT-PCR cannot distinguish between maternal and fetal infection. The absence of apparent abnormalities in these infants could be because 1) Zika virus may not have transferred from the mother to the fetus in utero because the late-pregnancy placenta might have protected the fetus, or an effective fetal immune response might be present; or 2) the critical period of organogenesis was complete before maternal or possibly fetal infection; therefore, no apparent/major malformations were identified at the time of birth. Clinical implications for an infant with Zika virus RNA detected in the placenta, in the absence of laboratory evidence of Zika virus in the infant, are unknown. However, the negative Zika virus testing results (cord blood or serum RT-PCR and serology) in these infants should be interpreted with caution; the results could be negative because the window of Zika virus detection might have passed. Periodic monitoring of these infants may be helpful for early recognition of potential late sequelae of congenital infections. Two recent studies observed neurologic abnormalities in infants whose mothers acquired Zika virus infection in the third trimester of pregnancy; for 1 of these infants, neurologic abnormalities were first identified at 6 months of age (*7**,**40*)*.*

In conclusion, our findings further support the linkage of Zika virus with microcephaly, suggest its association with adverse pregnancy outcomes, and demonstrate evidence of Zika virus replication and persistence in fetal brain and placenta. This article highlights the value of tissue analysis to expand opportunities to diagnose Zika virus congenital and pregnancy-associated infections and to enhance the understanding of mechanism of Zika virus intrauterine transmission and pathogenesis. In addition, the tissue-based RT-PCRs extend the time frame for Zika virus detection and particularly help to establish a diagnosis retrospectively, enabling pregnant women and their healthcare providers to identify the cause of severe microcephaly or fetal loss.

## References

[1] Hennessey M, Fischer M, Staples JE. Zika virus spreads to new areas—region of the Americas, May 2015–January 2016. MMWR Morb Mortal Wkly Rep. 2016;65:55–8. 10.15585/mmwr.mm6503e1 26820163

[2] Rasmussen SA, Jamieson DJ, Honein MA, Petersen LR. Zika virus and birth defects—reviewing the evidence for causality. N Engl J Med. 2016;374:1981–7. 10.1056/NEJMsr1604338 27074377

[3] Cauchemez S, Besnard M, Bompard P, Dub T, Guillemette-Artur P, Eyrolle-Guignot D, et al. Association between Zika virus and microcephaly in French Polynesia, 2013-15: a retrospective study. Lancet. 2016;387:2125–32. 10.1016/S0140-6736(16)00651-6 26993883 PMC4909533

[4] Simeone RM, Shapiro-Mendoza CK, Meaney-Delman D, Petersen EE, Galang RR, Oduyebo T, et al.; Zika and Pregnancy Working Group. Possible Zika virus infection among pregnant women–United States and Territories, May 2016. MMWR Morb Mortal Wkly Rep. 2016;65:514–9. 10.15585/mmwr.mm6520e1 27248295

[5] Calvet G, Aguiar RS, Melo AS, Sampaio SA, de Filippis I, Fabri A, et al. Detection and sequencing of Zika virus from amniotic fluid of fetuses with microcephaly in Brazil: a case study. Lancet Infect Dis. 2016;16:653–60. 10.1016/S1473-3099(16)00095-5 26897108

[6] Sarno M, Sacramento GA, Khouri R, do Rosário MS, Costa F, Archanjo G, et al. Zika virus infection and stillbirths: a case of hydrops fetalis, hydranencephaly and fetal demise. PLoS Negl Trop Dis. 2016;10:e0004517. 10.1371/journal.pntd.0004517 26914330 PMC4767410

[7] Brasil P, Pereira JP Jr, Raja Gabaglia C, Damasceno L, Wakimoto M, Ribeiro Nogueira RM, et al. Zika virus infection in pregnant women in Rio de Janeiro—preliminary report. N Engl J Med. 2016 Mar 4 [Epub ahead of print].;NEJMoa1602412. 10.1056/NEJMoa1602412 26943629 PMC5323261

[8] Tang H, Hammack C, Ogden SC, Wen Z, Qian X, Li Y, et al. Zika virus infects human cortical neural progenitors and attenuates their growth. Cell Stem Cell. 2016;18:587–90. 10.1016/j.stem.2016.02.016 26952870 PMC5299540

[9] Cugola FR, Fernandes IR, Russo FB, Freitas BC, Dias JL, Guimarães KP, et al. The Brazilian Zika virus strain causes birth defects in experimental models. Nature. 2016;534:267–71. 27279226 10.1038/nature18296 PMC4902174

[10] Li C, Xu D, Ye Q, Hong S, Jiang Y, Liu X, et al. Zika virus disrupts neural progenitor development and leads to microcephaly in mice. Cell Stem Cell. 2016;19:120–6. 10.1016/j.stem.2016.04.017 27179424

[11] Garcez PP, Loiola EC, Madeiro da Costa R, Higa LM, Trindade P, Delvecchio R, et al. Zika virus impairs growth in human neurospheres and brain organoids. Science. 2016;352:816–8. 10.1126/science.aaf6116 27064148

[12] Quicke KM, Bowen JR, Johnson EL, McDonald CE, Ma H, O’Neal JT, et al. Zika virus infects human placental macrophages. Cell Host Microbe. 2016;20:83–90. 10.1016/j.chom.2016.05.015 27247001 PMC5166429

[13] Bayer A, Lennemann NJ, Ouyang Y, Bramley JC, Morosky S, Marques ET Jr, et al. Type III interferons produced by human placental trophoblasts confer protection against Zika virus infection. Cell Host Microbe. 2016;19:705–12. 10.1016/j.chom.2016.03.008 27066743 PMC4866896

[14] Miner JJ, Cao B, Govero J, Smith AM, Fernandez E, Cabrera OH, et al. Zika virus infection during pregnancy in mice causes placental damage and fetal demise. Cell. 2016;165:1081–91. 10.1016/j.cell.2016.05.008 27180225 PMC4874881

[15] Adibi JJ, Zhao Y, Cartus AR, Gupta P, Davidson LA. Placental mechanics in the Zika-microcephaly relationship. Cell Host Microbe. 2016;20:9–11. 10.1016/j.chom.2016.06.013 27414496 PMC12606674

[16] Aliota MT, Caine EA, Walker EC, Larkin KE, Camacho E, Osorio JE, et al. Characterization of lethal Zika virus infection in AG129 mice. PLoS Negl Trop Dis. 2016;10. 10.1371/journal.pntd.0004682 PMC4836712 27093158

[17] Pulvers JN, Bryk J, Fish JL, Wilsch-Bräuninger M, Arai Y, Schreier D, et al. Mutations in mouse Aspm (abnormal spindle-like microcephaly associated) cause not only microcephaly but also major defects in the germline. Proc Natl Acad Sci U S A. 2010;107:16595–600. 10.1073/pnas.1010494107 20823249 PMC2944708

[18] Martines RB, Bhatnagar J, Keating MK, Silva-Flannery L, Muehlenbachs A, Gary J, et al. Notes from the Field: evidence of Zika virus infection in brain and placental tissues from two congenitally infected newborns and two fetal losses—Brazil, 2015. MMWR Morb Mortal Wkly Rep. 2016;65:159–60. 10.15585/mmwr.mm6506e1 26890059

[19] Martines RB, Bhatnagar J, de Oliveira Ramos AM, Davi HP, Iglezias SD, Kanamura CT, et al. Pathology of congenital Zika syndrome in Brazil: a case series. Lancet. 2016;388:898–904. 10.1016/S0140-6736(16)30883-2 27372395

[20] Mlakar J, Korva M, Tul N, Popović M, Poljšak-Prijatelj M, Mraz J, et al. Zika virus associated with microcephaly. N Engl J Med. 2016;374:951–8. 10.1056/NEJMoa1600651 26862926

[21] Driggers RW, Ho CY, Korhonen EM, Kuivanen S, Jääskeläinen AJ, Smura T, et al. Zika virus infection with prolonged maternal viremia and fetal brain abnormalities. N Engl J Med. 2016;374:2142–51. 10.1056/NEJMoa1601824 27028667

[22] Kourtis AP, Read JS, Jamieson DJ. Pregnancy and infection. N Engl J Med. 2014;370:2211–8. 10.1056/NEJMra1213566 24897084 PMC4459512

[23] Lanciotti RS, Kosoy OL, Laven JJ, Velez JO, Lambert AJ, Johnson AJ, et al. Genetic and serologic properties of Zika virus associated with an epidemic, Yap State, Micronesia, 2007. Emerg Infect Dis. 2008;14:1232–9. 10.3201/eid1408.080287 18680646 PMC2600394

[24] Karwowski MP, Nelson JM, Staples JE, Fischer M, Fleming-Dutra KE, Villanueva J, et al. Zika virus disease: a CDC update for pediatric health care providers. Pediatrics. 2016;137:pii:e20160621. 10.1542/peds.2016-0621 27009036

[25] Rabe IB, Staples JE, Villanueva J, Hummel KB, Johnson JA, Rose L, et al.; MTS. Interim guidance for interpretation of Zika virus antibody test results. MMWR Morb Mortal Wkly Rep. 2016;65:543–6. 10.15585/mmwr.mm6521e1 27254248

[26] Bhatnagar J, Blau DM, Shieh WJ, Paddock CD, Drew C, Liu L, et al. Molecular detection and typing of dengue viruses from archived tissues of fatal cases by rt-PCR and sequencing: diagnostic and epidemiologic implications. Am J Trop Med Hyg. 2012;86:335–40. 10.4269/ajtmh.2012.11-0346 22302871 PMC3269289

[27] Callahan JD, Wu SJ, Dion-Schultz A, Mangold BE, Peruski LF, Watts DM, et al. Development and evaluation of serotype- and group-specific fluorogenic reverse transcriptase PCR (TaqMan) assays for dengue virus. J Clin Microbiol. 2001;39:4119–24. 10.1128/JCM.39.11.4119-4124.2001 11682539 PMC88496

[28] Lanciotti RS, Kosoy OL, Laven JJ, Panella AJ, Velez JO, Lambert AJ, et al. Chikungunya virus in US travelers returning from India, 2006. Emerg Infect Dis. 2007;13:764–7. 10.3201/eid1305.070015 17553261 PMC2738459

[29] Nakajima N, Hata S, Sato Y, Tobiume M, Katano H, Kaneko K, et al. The first autopsy case of pandemic influenza (A/H1N1pdm) virus infection in Japan: detection of a high copy number of the virus in type II alveolar epithelial cells by pathological and virological examination. Jpn J Infect Dis. 2010;63:67–71. 20093768

[30] Wang F, Flanagan J, Su N, Wang LC, Bui S, Nielson A, et al. RNAscope: a novel in situ RNA analysis platform for formalin-fixed, paraffin-embedded tissues. J Mol Diagn. 2012;14:22–9. 10.1016/j.jmoldx.2011.08.002 22166544 PMC3338343

[31] Nowakowski TJ, Pollen AA, Di Lullo E, Sandoval-Espinosa C, Bershteyn M, Kriegstein AR. Expression analysis highlights AXL as a candidate Zika virus entry receptor in neural stem cells. Cell Stem Cell. 2016;18:591–6. 10.1016/j.stem.2016.03.012 27038591 PMC4860115

[32] Lazar M, Perelygina L, Martines R, Greer P, Paddock CD, Peltecu G, et al. Immunolocalization and distribution of rubella antigen in fatal congenital rubella syndrome. EBioMedicine. 2015;3:86–92. 10.1016/j.ebiom.2015.11.050 26870820 PMC4739417

[33] Ritter JM, Martines RB, Zaki SR. Zika virus: pathology from the pandemic. Arch Pathol Lab Med. 2016 Oct 5 [Epub ahead of print]. 10.5858/arpa.2016-0397-SA 27704909

[34] Halstead SB. Antibody, macrophages, dengue virus infection, shock, and hemorrhage: a pathogenetic cascade. Rev Infect Dis. 1989;11(Suppl 4):S830–9. 10.1093/clinids/11.Supplement_4.S830 2665015

[35] Winchester SA, Varga Z, Parmar D, Brown KE. Persistent intraocular rubella infection in a patient with Fuchs’ uveitis and congenital rubella syndrome. J Clin Microbiol. 2013;51:1622–4. 10.1128/JCM.03239-12 23426927 PMC3647901

[36] O’Neill JF. The ocular manifestations of congenital infection: a study of the early effect and long-term outcome of maternally transmitted rubella and toxoplasmosis. Trans Am Ophthalmol Soc. 1998;96:813–79. 10360309 PMC1298415

[37] Appler KK, Brown AN, Stewart BS, Behr MJ, Demarest VL, Wong SJ, et al. Persistence of West Nile virus in the central nervous system and periphery of mice. PLoS One. 2010;5:e10649. 10.1371/journal.pone.0010649 20498839 PMC2871051

[38] Bhatnagar J, Guarner J, Paddock CD, Shieh WJ, Lanciotti RS, Marfin AA, et al. Detection of West Nile virus in formalin-fixed, paraffin-embedded human tissues by RT-PCR: a useful adjunct to conventional tissue-based diagnostic methods. J Clin Virol. 2007;38:106–11. 10.1016/j.jcv.2006.11.003 17161650

[39] Labadie K, Larcher T, Joubert C, Mannioui A, Delache B, Brochard P, et al. Chikungunya disease in nonhuman primates involves long-term viral persistence in macrophages. J Clin Invest. 2010;120:894–906. 10.1172/JCI40104 20179353 PMC2827953

[40] Oliveira DB, Almeida FJ, Durigon EL, Mendes ÉA, Braconi CT, Marchetti I, et al. Prolonged shedding of Zika virus associated with congenital infection. N Engl J Med. 2016;375:1202–4. 10.1056/NEJMc1607583 27653589

